# A Reliable and Simple Method for Fabricating a Poly(Dimethylsiloxane) Electrospray Ionization Chip with a Corner-Integrated Emitter

**DOI:** 10.3390/s150408931

**Published:** 2015-04-16

**Authors:** Xiang Qian, Jie Xu, Cilong Yu, Yan Chen, Quan Yu, Kai Ni, Xiaohao Wang

**Affiliations:** 1 Graduate School at Shenzhen, Tsinghua University, Shenzhen 518055, China; E-Mails: vincent_2090@163.com (J.X.); yu-cl12@mails.tsinghua.edu.cn (C.Y.); yu.quan@sz.tsinghua.edu.cn (Q.Y.); ni.kai@sz.tsinghua.edu.cn (K.N.); wang.xiaohao@sz.tsinghua.edu.cn (X.W.); 2 Shenzhen Institutes of Advanced Technology, Chinese Academy of Sciences, Shenzhen 518055, China

**Keywords:** microfluidic chip, multi-layer soft lithography, electrospray ionization

## Abstract

Monolithically integrated emitters have been increasingly applied to microfluidic devices that are coupled to mass spectrometers (MS) as electrospray ionization sources (ESI). A new method was developed to fabricate a duplicable structure which integrated the emitter into a poly(dimethylsiloxane) chip corner. Two photoresist layers containing a raised base which guaranteed the precise integration of the electrospray tip emitter and ensured that the cutting out of the tip exerted no influence even during repeated prototyping were used to ease the operation of the process. Highly stable ESI-MS performance was obtained and the results were compared with those of a commercial fused-silica capillary source. Furthermore, chip-to-chip and run-to-run results indicated both reliability and reproducibility during repeated fabrication. These results reveal that the proposed chip can provide an ideal ion source for MS across many applications, especially with the perspective to be widely used in portable MS during on-site analysis.

## 1. Introduction

Micro-fabricated devices have proven highly advantageous for manipulating small sample volumes, integrating complex and diverse sample pre-treatments and separations, and coupling with other rapid detection methods. Integrated with pre-treatment modules and coupled with a mass spectrometer (MS), microfluidic chips can be applied in various kinds of quantitative analysis, such as biological, environmental, food safety and so on [1,2,3,4]. Several approaches to interface the microchips with an electrospray ionization mass spectrometer (ESI-MS) have been proposed [5]. The early approach emerging during the last decade was to fabricate open micro-channels directly at the edge of the chip. Although simple, the position of the Taylor cone along the chip edge was often unstable and difficult to control. The second approach which involved inserting the fused-silica capillaries directly into the ends of the channels was technically difficult to realize. In contrast, monolithically integrated emitters have been considered to be the most likely choice for microfluidic-based ESI-MS systems. However, the integrated emitters were mainly based on hard materials such as glass and silicon [6,7,8,9,10,11,12,13], so that the fabrication process for a single emitter base, given the required processes of lithography and etching, were always complicated, time-consuming, and expensive.

As an ideal alternative for monolithically integrated emitters, poly(dimethylsiloxane) (PDMS) had been taken into consideration in more and more experiments [14,15,16]. A polymer ESI microchip with an open tip emitter cast using a nickel mould which in turn was replicated from a dry-etched silicon wafer was reported [17]. A cutting process on a PDMS chip [18] or on a thin micro-channel embedded PDMS membrane [19] and a scrape process using a piece of curved transparency film [20] were also applied to form these emitters. Although these applications adopted soft lithography based on the PDMS into their fabrication, the processes were still complicated and the reproducibility of the structure under normal laboratory conditions needed improvement.

In the work reported here, we propose a simple, repeatable method for fabricating polymer microchips which directly lead the micro-channel to the corner of the microchip to form a corner-integrated emitter. This method retains as much simplicity as possible and improves the reliability of the fabrication process. The proposed method is based on standard soft lithography technology alone, but in the master fabrication procedure we carried out two exposures on two different photoresist layers [21]: this guaranteed a raised layer as the cutting base for precise integration of the electrospray tip emitter. Different from a series of significant works by Smith *et al*. [4,18,19], in which a direct cutting process was adopted based on a line marker, the raised layer-based cutting ensures the cutting process is much more stable. Due to the simple and repeatable fabrication processes and ease of integration with other PDMS-based microfluidic modules, the proposed ESI chip promises to be suitable as an ion source interface for a portable system. In the current research, we adopted a commercial ion trap mass spectrometer to test the performance of the ESI chip; but in the future we intend to integrate this chip with a portable MS under development by our group. Such portable systems, as reported by Cooks *et al*. [22,23,24], are promising for on-site applications such as detection of illegal food additives and environmental monitoring. We demonstrate how this microfluidic chip could be coupled to a mass spectrometer and achieve a better performance than a commercial fused-silica capillary system. The capability of mass fabrication and the reliability of reproduction by cast molding under normal laboratory conditions had huge potential to be widely used in portable MS instrument to facilitate on-site analysis.

## 2. Materials and Methods

### 2.1. Materials and Instruments

The master for the microchip was made of SU-8 negative photoresist (Microchem Corp., Naton, MA, USA) and chromium mask (Qingyi Precision Mask Making Co., Ltd., Shenzhen, China) was used in UV exposure. Microchips were made by mixing PDMS pre-polymer and curing agent (Sylgard 184, Dow Corning, Midland, MI, USA), and treated by oxygen plasma (PDC-M, Chengdu Mingheng Science & Technology Co., Ltd., Chengdu, China) before bonding. XYZ-manipulators (Beijing Optical Century Instrument Co., Ltd., Beijing, China) were used both in chip fabrication and performance experiments. A power supply module (Dongwen High Voltage Power Supply Co., Ltd., Tianjin, China), a pneumatic pressure controller (MFCS, Fluigent, Paris, France), a flowmeter (MFCS, Fluigent, Paris, France) and a short stainless steel needle (0.6 mm od and 0.1 mm id, Shanghai Tianyou Rubber and Hardware Co., Ltd., Tianjin, China) were used in the experiments. Rhodamine B (Dr. Ehrenstorfer GmbH, Augsburg, Germany), hexamethyldisilylamine (HMDS, Aladdin Inc., Los Angeles, CA, USA), methanol and acetic acid (Merck KGaA, Darmstadt, Germany) used in the experiments were HPLC grade. An ion trap mass spectrometer (Thermo Fisher Scientific Inc., Waltham, MA, USA) was coupled with the microchip and MS data were collected by the computer. Photographs of the electrospray plume were captured using a CCD camera under the laser (MBL-H-480, Changchun New Industries Optoelectronics Tech Co., Ltd., Changchun, China).

### 2.2. Microchip Design and Fabrication

**Figure 1:**
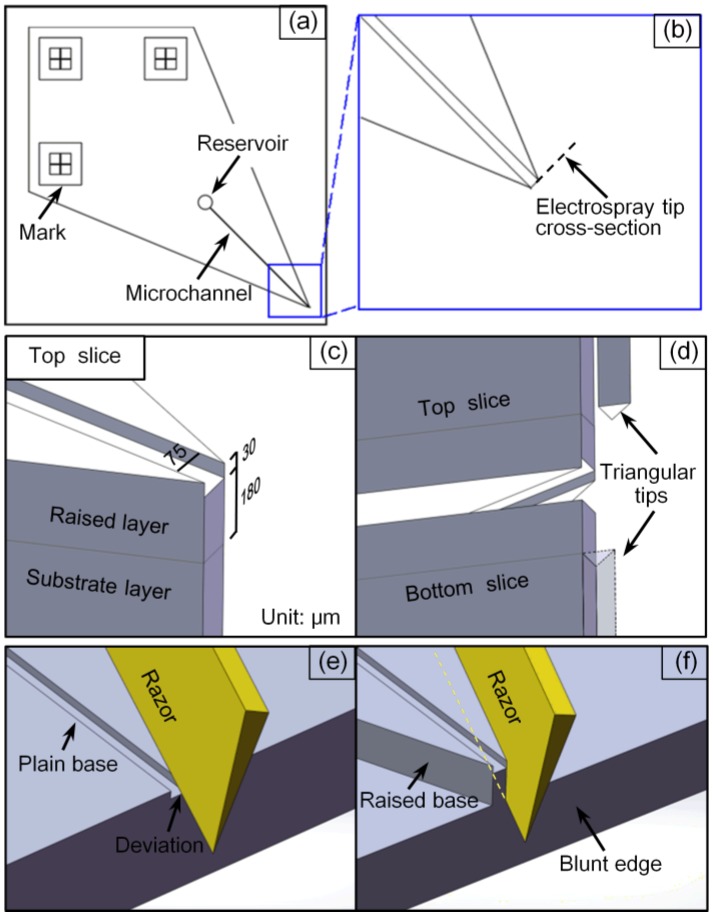
Diagrams of the chip’s design and fabrication. (**a**) AutoCAD design of the microfluidic emitter-integrated chip; (**b**,**d**) blunt configuration for the chip corner tip emitter, eliminating the influence of triangular tips in top and bottom slices; (**c**) dimensions of the top slice; (**e**,**f**) comparison between plain and raised bases for chip cutting: there will be deviations arising from the use of a plain base.

This simple, repeatable, microfluidic chip with a corner-integrated emitter was fabricated based on PDMS: as shown in Figure 1a (its diamondoid schematic), a micro-channel of 20 mm in length stretched from a reservoir of 2 mm in diameter directly to the sharp corner at an angle of 45°, similar to the design of a glass microchip proposed by Ramsey [25]. There were also three marks composed of crosses and squares in the chip, used for alignment during fabrication. The channel had a full width of approximately 75 μm and a depth of 30 μm, and lay on a base of 180 μm in height (Figure 1c). Moreover, the end of the micro-channel (Figure 1b,c), namely the electrospray tip emitter, had a blunt configuration.

**Figure 2:**
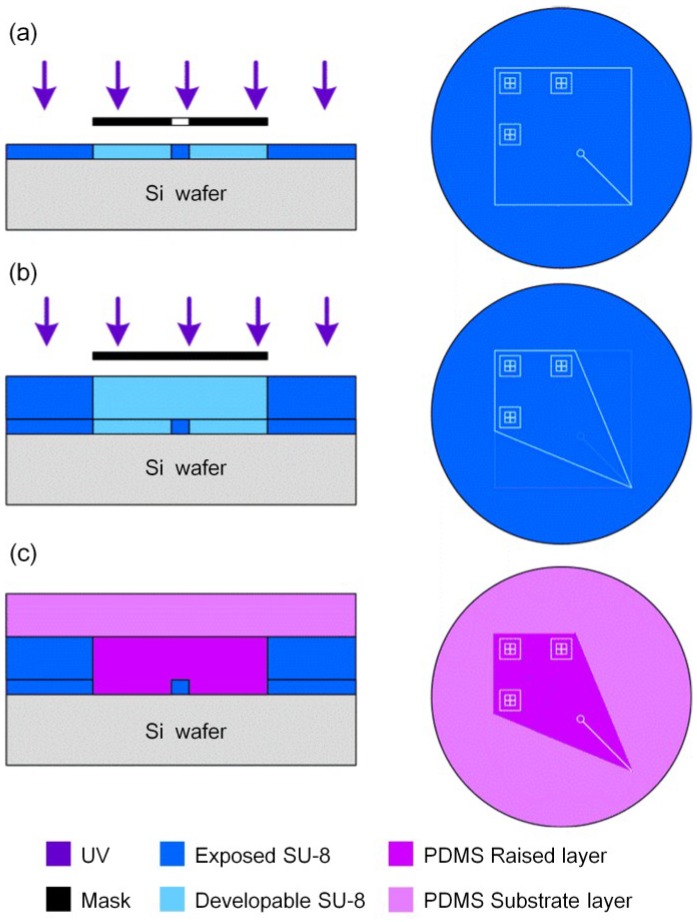
Process schematic. (**a**) the first *ca*. 30 μm thick SU-8 photoresist layer was subjected to UV exposure; (**b**) the second *ca*. 180 μm photoresist layer was subjected to UV exposure with another mask; (**c**) PDMS pre-polymer was poured onto the photoresist master and the raised and substrate layers were both formed.

This microfluidic chip was fabricated by standard soft lithography [26], but it needed two masks and two corresponding layers of photoresist material to realize its design. Briefly, the main procedure was as follows: (1) in Figure 2a, after the first *ca.* 30 μm thick SU-8 photoresist layer was spun, one chromium mask containing transparent areas of the marks, reservoir, and micro-channel was subjected to UV exposure. This mask had a slightly larger outline than the diamondoid chip. The marks were transferred onto the photoresist layer, which would be used to align these structures to those in another mask; (2) in Figure 2b, the second photoresist layer was spun to *ca.* 180 μm in thickness, and the other mask was used to form the diamondoid outline. Most importantly, the end of the micro-channel was exactly located at the sharp corner, aligned by using the XYZ-manipulator; (3) in Figure 2c, the above two steps formed the needed master after photoresist development. The PDMS pre-polymer and curing agent were mixed in ratios of 5:1 and 20:1 for the top and bottom elastomer slices, respectively; this can facilitate the formation of a hermetic seal between them [27]. Then the mixture was poured onto the master and post-cured in an oven at 80 °C for 1 h. The diamonded raised layer was then shaped with the micro-channel lying upon it, and the raised layer was supported by the substrate layer. The substrate layer made it easy to peel the whole PDMS layers off the master.

Consequently, the raised layer was quite thin compared to the substrate layer so it was necessary to cut off the redundant part of the substrate layer in case the spray was hindered thereby. The edge line of the razor was manually placed over the bottom line of the raised layer and cut in (Figure 1f). There were three cuts consisting of the two edges of the 45° angle and the blunt channel end. This micro-channel contained slice was the top one of the chip and the bottom one was fabricated in the same way except that its photoresist master only needed one-time lithographic processing as the second step, to form the diamondoid embossment corresponding to its upper counterpart. After separate curing and cutting, both PDMS slices were treated in oxygen plasma followed by a bonding procedure on a XYZ-manipulator.

### 2.3. Mass Spectrometry

**Figure 3:**
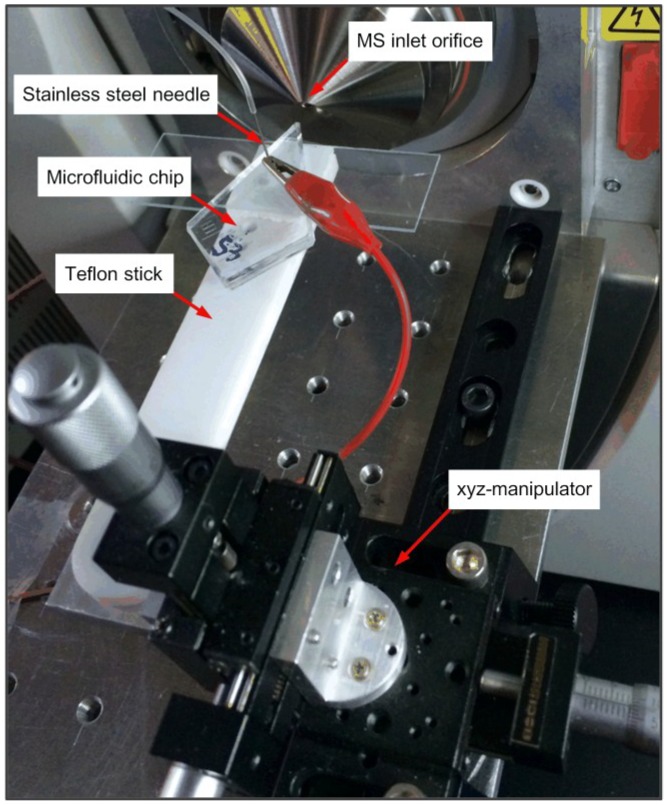
Experiment platform. A laboratory-built platform was set up on the commercial ion trap MS to perform the microchip experiments.

This diamondoid chip was further subjected to a hydrophobic treatment before the experiments, which was done by treating the bonded microchip with volatilized hexamethyldisilylamine (HMDS) gas in a closed vessel, and it was then held by a laboratory-built platform coupled to the ion trap mass spectrometer, as illustrated in Figure 3. A Teflon stick placed the electrospray tip emitter several millimeters away from and aligned to the mass spectrometer inlet orifice: adjustment was undertaken by a XYZ-manipulator. The sample solution was conveyed to the reservoir through the short stainless steel needle embedded in the reservoir: this was controlled by the pneumatic pressure controller. A high voltage generated by the power supply module was applied via the needle to the solution to produce the required electro-kinetic force. In the ESI-MS coupling experiments, the distance and voltage between the electrospray tip and the MS orifice was significant with regard to its effects on MS signal stability and intensity. The optimum distance and voltage to be applied for current setup were determined experimentally as about 4 mm and +4.5 kV, respectively.

Comparison experiments were operated using the commercial fused-silica capillary source of the ion trap mass spectrometer. The outer diameter (od) and inner diameter (id) of the commercial fused-silica capillary for comparison were 0.19 mm and 0.10 mm, respectively, and its tip was not tapered.

## 3. Results and Discussion

### 3.1. Characteristics of the Chip Fabrication

**Figure 4:**
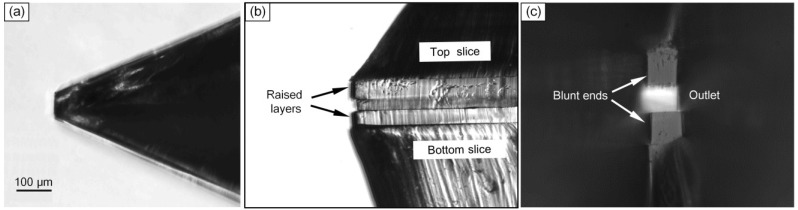
Micrographs of the corner integrated emitter. (**a**) Top view of the *c.* 75 μm blunt tip of the 45° corner; (**b**) Lateral view of the tip, the micro-channel layer lies between the two raised layers; (**c**) Front view of the tip: the blunt ends and outlet are clearly distinguishable.

This polymer microfluidic chip with the corner-integrated emitter was fabricated as described above. Its microscopy images are shown in Figure 4. The accurate and reproducible two-layer method realized the target structure’s production and the electrospray tip was sufficiently firm. Although the top and bottom slices were not aligned that accurately, they still formed an almost perfect outlet (Figure 4c).

The technology used in this work ensured that the end could already be blunt in the micro-channel contained layer and the 180 μm thickness raised layer when de-moulded (due to the lithography properties of the SU-8), and that the cuts only needed to be applied to the substrate layer without impacting the vulnerable channel. This optimized end, by further removing the tiny triangular tip of the substrate layer, was beneficial with regards to the elimination of the influence on the spray when there were tiny triangular tips protruding out of the electrospray tip cross-section of both top and bottom slices (Figure 1d). In the fabrication procedure of these ends, a raised base was formed in the lithography process and three manual cuts with a razor were needed for the substrate layer. The end was tiny, and the polymer was elastic, therefore if the blunt end was the last to be cut, there would be damage to the end. So the first cut was made to form the blunt edge and the other two were used for the angle’s two edges (Figure 1f). For the particular polymer properties, the cut-off PDMS parts could remain compressed and mutually affixed to the reserved part if just cut. This facilitated the latter cuts and only after all cuts had been made, were the cut-off parts removed. By moving against the side wall of the raised base, it was easy, when using a razor blade, to cut the substrate layer manually under an optical microscope. If the commonly used plain base was adopted, there would be deviations arising while cutting which would damage the channel (Figure 1e). Although the cutting edges on the substrate layer were not especially trim in this work, they would not affect the highly embedded micro-channel, even during repeated prototyping (Figure 1f).

During the fabrication of this chip, it was both quick and easy to accomplish and guarantee the alignment of the chip corner and micro-channel end and between the top and bottom slices. Moreover, the integration of the chip corner and the micro-channel end can be performed simultaneously before the PDMS elastomer was peeled off the master. Instead of cutting out the corner on the channel layer as is done when fabricating glass chips [28], this method overcame the problem of the elasticity of the PDMS elastomer. Compared to other methods [4,17,18,19,20,29], this structure lends itself to mass fabrication and sustained high reliability. There was also less equipment required, much less cost, and less time consumed.

**Figure 5:**
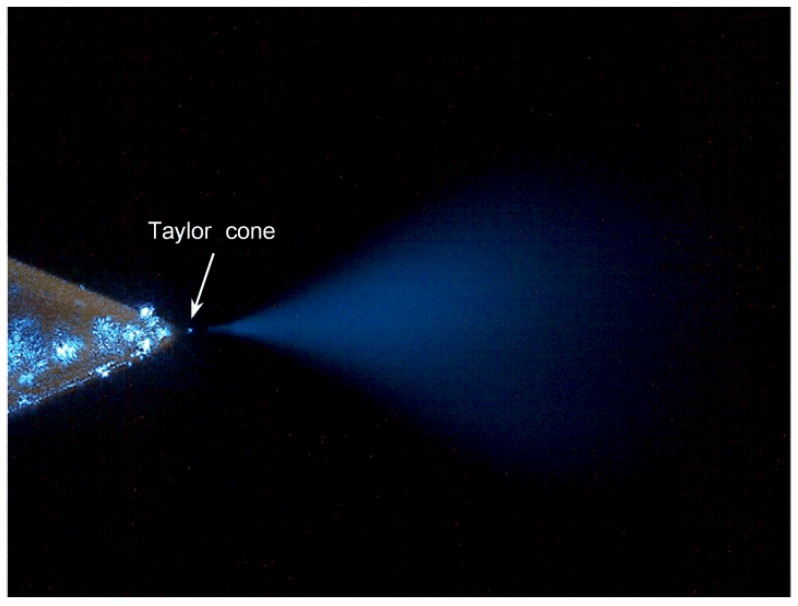
Image of the laser-illuminated electrospray: this is a frame captured from video footage of the electrospray plume generated from the corner integrated emitter tip.

The stability of this chip can be assessed by its electrospray plume. Viewed through an optical microscope, the electrospray plume can be clearly seen under illumination by a 10-mW blue laser. A frame captured from video footage of the laser-illuminated electrospray plume is shown in Figure 5. At the end of the sharp corner, we can observe the Taylor cone as a small bright spot, which remained steady and without visible change during the whole process as well as the plume spreading from the spray tip. The hydrophobic PDMS made the Taylor cone non-diffuse when it came into contact with the side wall around the outlet: this was superior to methods using disruptive hydrophilic materials such as glass [30,31].

According to the geometries of the chip, and under the given pressure from 0 to tens of mbar, the mean flow rate in the micro-channel was estimated at below several hundred nL/s [32]. We found that once the pneumatic driving pressure was set close to 0 mbar, the electro-kinetic force (mainly the electroosmotic flow in the micro-channel) alone cannot conquer the flow resistance of the micro-channel to produce a stable electrospray; on the other hand, much higher pneumatic driving pressure can also destroy the continuity and stability of the electrospray plume. We suggested that the electro-kinetic force in the electrospray process should keep in balance with the driving force generated by the pressure and the interaction between the channel and solution, which in fact was the balance between inflow and outflow. For our current setup, the pneumatic driving pressure should be set around 25 mbar, and the final mean flow rate was from about 1 μL/min to 10 μL/min.

### 3.2. Performance Tests

**Figure 6:**
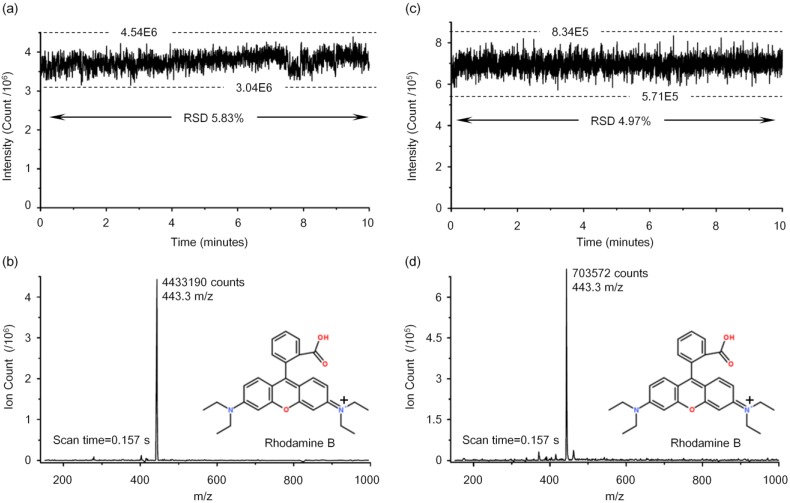
Data acquired while infusing 10 μM Rhodamine B in 50/50 (v/v) methanol/water containing 0.2% acetic acid at an injection pressure of 25 mbar. The electrospray potential was +4.5 kV. (**a**,**c**) Rhodamine B ion counts and signal stability of the proposed corner integrated emitter and the fused-silica capillary, respectively; (**b**,**d**) Single spectra over a 0.157 seconds scan time of the proposed corner integrated emitter and the fused-silica capillary, respectively.

In the current work, we selected Rhodamine B, a fluorescent dye and illegal food additive in spice sauces [33], as the sample to test the proposed ESI chip. In the ESI+ mode, the precursor ion is the rhodamine B cation (*m/z* = 443.3) without the Cl^−^ ion. A mixture of 10 μM Rhodamine B in 50/50 (v/v) methanol/water containing 0.2% acetic acid, which was infused at an injection pressure of 25 mbar, was firstly used as a standard sample to test the performance of this diamondoid chip. Initially, we removed bubbles from the channel and filled it with solution using the “purge” function of the pneumatic pressure controller: the test voltage was then applied. It was essential to slightly adjust the manipulator to ensure that the electrospray plume and the MS inlet orifice were coaxial. After the optimum signal of the Rhodamine B precursor ion was obtained, 10 min of mass spectral data were acquired (Figure 6). The stability of the electrospray signal is shown in Figure 6a. The relative standard deviation (RSD) for the Rhodamine B ion counts was 5.8%, the same level as that of a fused-silica capillary source in Figure 6c (5.0%), which was acquired under the same conditions. However, as indicated in Figure 6a, the average ion counts was at level of 10^6^ (3 × 10^6^ ~ 4 × 10^6^) for the proposed corner integrated emitter, higher than the fused-silica capillary (at level of 10^5^ in Figure 6c). Also indicated in Figure 6b,d, the comparison of single spectra over one scan time showed the better signal intensity and signal to noise ratio (*S/N*) of this device.

**Figure 7:**
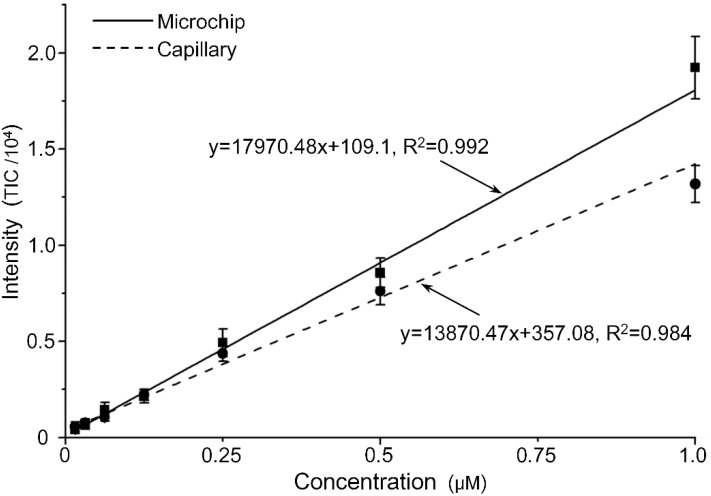
Different signal intensities of sample solutions in different concentrations. Each point represents the mean value of signal data and error bar for the deviation at each concentration.

To further demonstrate the stability and sensitivity of the proposed corner integrated emitter, experiments under the same experimental condition but at much lower concentrations and much lower flow rate were conducted. The concentrations of the Rhodamine B in 50/50 (v/v) methanol/water containing 0.2% acetic acid were: 0.015625, 0.03125, 0.0625, 0.125, 0.25, 0.5 and 1 μM and the sample solution were injected by the pneumatic pressure controller at a measured flow rate of 1.6 μL/min; the same flow rate under the same concentration for both the proposed chip and the capillary ensured the same amount of matter to compare the ESI performance of both setup. The results have been fitted in Figure 7, revealing that the overall linearity and sensitivity of both the setup were comparable or even slightly superiority for the proposed chip, such that the Rhodamine B intensity-concentration relationship of the proposed corner-integrated emitter had a higher slope (about 1.3 times) than those of the fused-silica capillary, which means that a smaller concentration increase can provide more intensity change, in other words, it is more sensitive; and the coefficient of determination (*R*^2^) for the fitted line of the proposed emitter (0.992) was also higher than that of the capillary (0.984), meaning that the linearity between intensity and concentration of the proposed chip was better. The limit of detection for Rhodamine B was estimated to be lower than 0.015 μM (signal intensity at ~10^2^, *S/N* = 19) for the current setup. The *S/N* results had also been calculated as 33, 66, 146, 173, 509 and 1065 for concentrations from 0.03125 μM to 1 μM, revealing the good sensitivity of this microchip.

**Figure 8:**
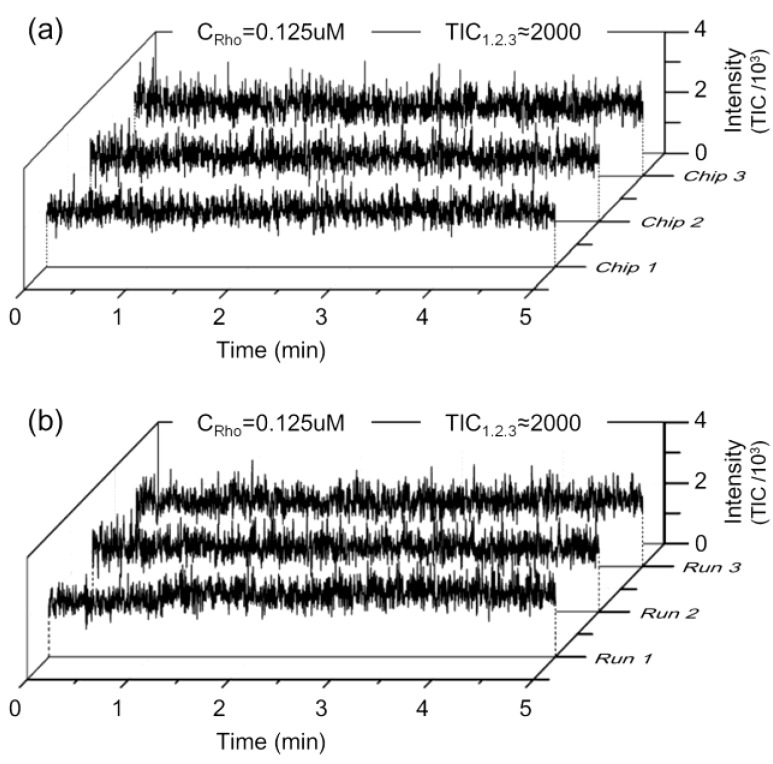
Reproducibility of chip-to-chip and run-to-run tests. (**a**) The mean ion counts of Rhodamine B were 1969.90, 2137.60 and 2055.85 for chips 1 to 3, respectively when the solution concentration was 0.125 μM; (**b**) the mean ion counts were also approximately 2000 for run 1 to 3 of chip 2. The solution concentration was 0.125 μM.

Furthermore, chip-to-chip and run-to-run reproducibility, which is of highly importance in analytical applications [34,35], have been tested as illustrated in Figure 8. The mean ion counts of Rhodamine B were 1969.90, 2137.60 and 2055.85 for chips 1 to 3, respectively, while the solution concentration was 0.125 μM (Figure 8a). At the same concentrations, a single chip in different test runs also showed excellent reproducibility (signal intensity around 2000), as shown in Figure 8b. All other concentrations selected in our experiment also indicated the same chip-to-chip and run-to-run relationship. These results verified the reliability and stability of the proposed chip during repeating fabrication.

Nowadays, the nanoelectrospray technique has attracted more and more attention among the cell biology and molecular biology research community, especially for peptide and protein analytes. From the flow rate point of view, it’s known that the nanoelectrospray regime or the microelectrospray regime are mainly decided by the size of the emitter outlet, and whether a “true” nanoelectrospray will present advantages under certain condition should be considered [36]. For example, in Smith’s work [4,18], the size of the channel and the emitter was 8 μm × 12 μm and the working flow rate was mainly 50 nL/min to 1 μL/min, and as reported by Kim *et al*. [20] it was 30 μm × 100 μm with a flow rate of about 1 μL/min to 20 μL/min. For on-site applications considering the stability and reproducibility and to avoid clogging problems, we selected a moderate size of 30 μm × 70 μm and the working flow rate was a little above 1 μL/min as in the microelectrospray regime. From the sensitivity point of view, on the other hand, comparing to other outstanding performances like the 1 nM reported in Smith’s work [4], the difference may not only be affected by the emitter but also the mass spectrometer and other conditions. We adopted the LCQ to verify our emitter as the commercial system most relevant to our final goal of manufacturing a portable ion trap mass spectrometer, where in Smith’s work [4], an ion funnel modified TOF was adopted; the sensitivity difference for the mass spectrometer alone may be of an order of magnitude. Although our total sensitivity was not the as remarkable as the previous one, the stability and reliability was verified and seemed to be salient according to the raised layer cutting process. Further optimization of the ion transport system, the mass spectrometer and the overall experiment setup will be adopted in our future work, in order to reveal the ultimate sensibility in the nanoelectrospray regime for the current PDMS emitter fabricated by the proposed process.

What could not be neglected is that the PDMS is a porous material, absorbing small molecules when solutions flow through channels and affecting the sensitivities. Although solvent extraction and surface modification can reduce this influence, the adsorption properties are closely related with the samples, which means different samples have different adsorption ratios. Rhodamine 6G had been reported to show obvious adsorption after a relatively long period of incubation time [37]. In consideration of that there’s no report about Rhodamine B’s adsorption at present and the residence time of solutions in the microchannel were quite short, future research should further verify the effect of Rhodamine B’s adsorption on the sensitivity.

Overall, these results verified the performance of the proposed corner-integrated emitter; the observed small superiority over the commercial fused-silica capillary source should mainly be due to the blunt end configuration, the hydrophobic properties of the PDMS and the further hydrophobic treatment for the blunt tip. The proposed chip was also shown to be reliable during repeated fabrication due to the simple multilayer soft lithography and raised layer based cutting.

## 4. Conclusions

In conclusion, the monolithically corner-integrated emitter described in this work demonstrated highly stable ESI-MS performance, as well as high signal intensity and high reproducibility. It was comparable to the performance of commercially available fused-silica capillary sources. The proposed fabrication steps were easily realized and after finishing the photoresist master by lithography, all other processes could be run under normal laboratory conditions. This ensured that the proposed corner integrated emitter was suitable for mass fabrication and it maintained its high reliability at a potentially lower cost. Integrated with other microfluidic based pre-treatment modules into a single chip, it provided an ideal ion source for MS across many applications, especially with the perspective to be widely used in portable MS instrument during on-site analysis.
